# Nowhere to Go: Parents' Descriptions of Children's Physical Activity During a Global Pandemic

**DOI:** 10.3389/fpubh.2021.642932

**Published:** 2021-04-26

**Authors:** Deanna Perez, Janelle K. Thalken, Nzubechukwu E. Ughelu, Camilla J. Knight, William V. Massey

**Affiliations:** ^1^Psychosocial Physical Activity Laboratory, Kinesiology Department, College of Public Health and Human Sciences, Oregon State University, Corvallis, OR, United States; ^2^School of Sport and Exercise Sciences, Swansea University, Swansea, United Kingdom

**Keywords:** COVID-19, health, families, children, active play

## Abstract

**Background:** Schools and outdoor public spaces play a substantial role in children's physical activity. Yet, the COVID-19 shelter-in-place mandates bound many children to their available home spaces for learning, movement, and development. The exact effect this mandate had on children's physical activity may vary among families.

**Objective:** To understand, from the perspective of parents, how the COVID-19 shelter-in-place mandates affected children's physical activity, while also considering families' socioeconomic status.

**Design:** Open-ended survey.

**Setting:** Online.

**Method:** Data were collected from 321 parents living in the United States of America. Parents answered an open-ended prompt to describe their children's physical activity during COVID-19 shelter-in-place mandates. Following data collection, inductive and deductive content analysis examined patterns in the data.

**Results:** Analyses indicated that shelter-in-place mandates restricted children's opportunities for physical activity. However, if families had access to outdoor spaces or equipment, they could encourage and support more physical activity opportunities than those without. Families in the lower-income bracket had less access to outdoor space and subsequently those children had fewer opportunities to be physically active. Parents supported their children's physical activity through their involvement and encouragement.

**Conclusion:** These findings underscore the importance of access to outdoor spaces and equipment for increasing children's physical activity. Findings can be used by educators and policymakers to equitably support families of lower socioeconomic status who reported less access to outdoor spaces.

## Introduction

Physical activity (PA) is vital for children's health and development (1). Based on current PA guidelines, children should engage in 60 min of moderate to vigorous PA (MVPA) each day (1), yet only 24% of children and youth are meeting current PA guidelines (2). A systematic review makes it evident that children's sedentary behavior is negatively associated with outdoor physical activity (3). Children engage in more PA outdoors compared to indoors (4–6).

Children in urban and low-income communities have less access to recreation and PA due to a number of environmental influences. Specifically, barriers to PA among urban youth include high levels of community violence, financial burdens related to extracurricular activities, lack of organized programs, scarcity of green spaces, and unsafe spaces (7, 8). Compared to youth in mostly White, affluent areas, youth in low-income communities of color are less likely to live near recreational facilities and more likely to be obese (9).

In 2020, public health and government officials directed the coronavirus disease-2019 (COVID-19) shelter-in-place mandates to prevent the transmission and acquisition of the coronavirus among Americans. As a result, schools, public parks, and summer camps closed across the United States of America (USA), thus hindering outdoor physical activity. The COVID-19 shelter-in-place mandates bound many children to their available home spaces for learning, movement, and development. The exact effect of this mandate on children's PA may vary among families. Additionally, parents have not only had to manage their own workloads, many of which have changed, but they have also had to take on additional roles and responsibilities, including facilitating children's at-home education. Consequently, the nature of parents' workload and the time they could commit to their children throughout this period may also influence PA.

Practitioners and public health officials strive to support families in ways that enhance children's PA and health. Stakeholders work to increase PA during the school day, after school, and throughout summer breaks (10). Policymakers consider how to best support a health-promoting built environment and active transportation (11). To support effective public health initiatives, scientific research should appropriately consider the constraints and restrictions experienced by families, including those in different communities and of various socioeconomic statuses. Public health interventions can be more effective and appropriately adapted when researchers more fully understand the nuanced PA experiences of children and families of various backgrounds. Moreover, identifying the factors that influence children's PA during the COVID-19 shelter-in-place mandates can also provide pertinent insight into factors that could enhance children's PA outside of the school environment more generally. Therefore, the purpose of the current study was to understand, from the perspective of parents, how the shelter-in-place mandates affected children's PA, while also considering families' socioeconomic status.

## Methods

This study was exploratory, and the purpose required an approach that would enable a descriptive insight into parents' perceptions of children's PA during unprecedented times. As such, a qualitative descriptive approach was utilized (12, 13). Qualitative description studies can be used to provide a descriptive and detailed examination of events—in this case PA in a pandemic (12). Additionally, qualitative description is particularly appropriate when seeking to obtain answers to questions that have implications for practitioners and policymakers (12). Qualitative description is guided by the basic tenets of naturalistic inquiry.

### Participants

Participants were eligible to complete the survey if they were 18 years of age or older and the parent or guardian of at least one child in primary or secondary school, which is kindergarten through 12th grade in the USA. To collect a wide spectrum of experiences about how the shelter-in-place mandates affected families of different socioeconomic statuses, data collection was stratified by income bracket.

### Data Collection

Data were collected through the online platform Prolific, which is designed for recruiting research participants [www.prolific.co (14)]. Through Prolific, researchers can recruit pre-screened participants and aim to collect data from select samples. Prolific has been shown to produce high quality data with diverse participants (15). Prolific conducts quality checks limiting random responses and bot accounts. These quality checks include, but are not limited to, verifying phone numbers, limiting number of accounts using the same internet protocol (IP) address or internet service provider (ISP), restricting account sign up based on IP and ISP, investigating suspicious accounts reported by researchers, and monitoring unusual usage patterns *via* internal data analysis (16). Prior to accessing the survey, participants gave informed consent and agreed to participate by clicking the survey link. Upon completion of the survey, participants were paid $1.75 USD. Participants were asked to respond to an open-ended prompt and “describe [their] children's level of physical activity during COVID-19 shelter-in-place mandates.” (See Appendix for survey questions.) The COVID-19 pandemic has impacted different regions of the USA at different times; thus, it is important to note that data collection occurred in April and May of 2020.

### Data Analysis

Grounded in a social constructionist approach to inquiry, data were analyzed using content analysis. In the first stage of analysis, the entire dataset was analyzed. Researchers coded raw data *in vivo*, organized codes by larger patterns, and came to a consensus to develop four core themes (17). In the second stage of analysis, researchers separated data based on household income levels. Specifically, researchers examined data from families who reported an annual household income of < $45,000 USD and those families with an annual household income >$95,000 USD. This part of analysis was deductive. Researchers sought to determine if the themes presented themselves differently depending on families' socioeconomic status.

## Results

### Demographic Information

The 321 research participants fell into each of the following three annual household income brackets: (1) < $45,000 United States Dollar (USD; 24%), (2) between $45,000 and $94,999 USD (45%), and (3) more than $95,000 USD (32%). Participants identified as White (81%), Latinx (6%), Asian (5%), Black (5%), or “Other” (3%). (See Table 1 for more data on the sample). Most participants (*n* = 314) described themselves as parents, but five participants identified as stepparents and two participants identified as grandparents.^1^

**Table 1:** Demographic information of parents (*n* = 321) and children (*n* = 493) in the sample.

**Category**	**Variables**	**Percentage/Proportion**
Parents' race	White	81.3/260
	Latinx	6.3/20
	Asian American	5.1/17
	Black	4.5/15
	Native American	0.2/1
	Other	2.8/8
Household income (USD)	Less than 45K (Low income)	24.0/77
	45K−94,999	44.5/143
	Above 95K (High income)	31.5/101
Children per family	Parent with 1 child	55.5/178
	Parent with 2 children	34.3/110
	Parent with 3 children	9.0/29
	Parent with 4 children	0.9/3
	Parent with 5 children	0.3/1
Parent in income group with more than 1 child	Low income High income group	27 52
Parental status	Mother Father Parent^a^^,^^b^ Grandparent	50.8/163 32.1/103 16.5/53 0.6/2
Qualified for free or reduced-price lunch^c^	Yes No	28.0/90 71.7/230 9.8/3.6
Children's average age/SD (years) Children with disabilities	Developmental Physical Behavioral/Emotional	7.8/38 2.2/11 11.4/56
Gender	Male Female	52.9/261 47.1/232

*Missing data were recorded in the following category: race (1), income (2), and (3) free/reduced lunch*.

a *Participants were given the option to identify as parent without specifying if they were the mother or father*.

b *Five participants identified as stepparents to at least one of their children*.

c *US families with a lower household income can qualify for free or reduced-price lunches at K−12 schools through a federally assisted meal program*.

### Restricted Physical Activity Opportunities

Following data analysis, four overall themes were identified (See Table 2 for more details and quotations). The first theme was *shelter-in-place mandates restricted children's opportunities for physical activity*. Overall, the shelter-in-place mandates coincided with parents reporting that their children had fewer opportunities to be physically active. This theme was true for families regardless of their reported household income. The shelter-in-place mandates imposed new restrictions for PA, such as closed playgrounds, while also amplifying existing disparities in access to PA. One mother shared, “My child is less active during the mandates. We try to get out and play, but playgrounds have been closed, and the weather is too hot outside.”

**Table 2:** Parents describe children's PA during COVID-19 shelter-in-place mandates.

**Theme**	**Bracket**	**Differences by income bracket^a^**	**Quotation**
*Shelter-in-place mandates restricted children's opportunities for PA*	Lower income	No differences.	*Less than normal because they can't see their friends, but we still try to incorporate it into the day*.
	Higher income		*Has been very much curtailed. Child doesn't have anyone else to really play with*. *Not as active as normal. No soccer or swimming*.
*Access to outdoor activity spaces*			
	Lower income	Less access to outdoor spaces and less reported outdoor PA.	*Not as much [PA]. The house is small, but they still get it in*. *My child is less active during the mandates. We try to get out and play, but playgrounds have been closed*. *My child is less physically active, but still practices indoor activities like dancing and yoga*.
	Higher income	More access to outdoor spaces and more reported outdoor PA.	*We go outside as often as possible. My kids typically run and play in our yard. I also take them to open spaces in city parks where we throw frisbees and walk through nature. We also ride scooters and take walks around the neighborhood often*. *Our children have been able to play in the backyard. We have a gated driveway that goes to the back of the house, so they have been able to play games like four square, basketball, scooter, and ride bikes. They have spent quite a bit of time outside*.
*Access to exercise and play equipment*			
	Lower income	No differences.	*My child is normally very active riding his hover-board, playing with the dogs, or jumping on the trampoline*. *We use my exercise bike for 10 minutes each day, 10 more minutes of yoga, and multiple dance parties each day to help break up the monotony and stop anxiety when it starts*.
	Higher income		*Have made several purchases of playground equipment and a trampoline to encourage outside activity during shelter in place measures*.
*Parents support their children's PA*			
	Lower income	No differences.	*We continue to encourage bike riding and walks, but it's not as high as pre-covid levels*. *My kids remain physically active, however they are usually being regulated their mom*.
	Higher income		*My child is engaging in active physical play throughout the day and every day after I get off from work we go for a family jog/walk*. *We have as a family picked up bike riding, taking the dogs for daily walks, basketball in the driveway along with four square, swimming and tennis*.

a *The lowest income bracket included households with a self-reported annual income of < $45,000 USD. The highest income bracket included households with a self-reported annual income >$95,000 USD*.

New restrictions include canceled youth sport seasons, dance classes, after-school programs, and in-person physical education (PE) classes. Children no longer had the opportunity to be active through those means. A mother described her 14-year-old son as becoming “more of a couch potato. Before COVID-19, he played on two basketball teams concurrently, so he was always going to a practice or game, and he also participated in after-school intramural sports 2 or 3 days a week. None of those team-sport activities can be safely done now.”

Another new restriction that families experienced was the lack of friends and peers available for their children to play with. A mother shared that her children's PA was “less than normal, because they can't see their friends, but we still try to incorporate it into the day.” Overall, parents reported keeping children socially distant from other children. Children played by themselves, with siblings, or with parents.

### Access to Outdoor Spaces

The second theme was *access to outdoor activity spaces*. Across all income brackets, when families had access to outdoor spaces, they also had the opportunity to engage in outdoor PA. Parents used outdoor spaces as a means of providing safe spaces for their children to be active. Examples of outdoor spaces include backyards, driveways, quiet streets, or sidewalk space. However, access to outdoor spaces was not the same for all income brackets. Families in the lower income bracket had less access to these outdoor spaces and reported less engagement in outdoor PA. A mother of a 5-year-old boy and 7-year-old girl, with a household income of < $20K, shared that there is “not much activity. We don't have a big yard to play in.” Families in the higher income bracket had more access to outdoor spaces and reported more outdoor PA. A father of two girls, with a household income >$120K, shared that his “children have been able to play in the backyard. We have a gated driveway that goes to the back of the house, so they have been able to play games like four square, basketball, scooter, and ride bikes. They have spent quite a bit of time outside.”

Furthermore, the shelter-in-place mandates restricted families' access to indoor PA spaces (such as school gyms, YMCAs, or Boys and Girls Clubs). A parent of a 10-year-old boy, with a household income $45–70K, lamented that “the parks are closed, and the basketball courts are closed. Hell, everything is closed.” Children have less opportunity to engage in PA when families already have restricted access to outdoor spaces (as is the case with families in the lower income bracket) and indoor PA spaces have become unavailable as well.

### Access to Equipment

The third theme across all income groups was *access to exercise and play equipment*. Analysis identified that the availability of equipment coincided with higher reports of engagement in PA. Within the sample, play and sport equipment promoted opportunity for children's PA. Examples of equipment included indoor exercise equipment, trampolines, sport equipment, bikes, and scooters. A parent, with a household income $45–70K, shared that their daughter “does not have access to outdoor equipment at home, and parks are closed.” A few parents mentioned purchasing new equipment for their children in an effort to promote PA. A father of two girls, with a household income >$120K, shared that he “made several purchases of playground equipment and a trampoline to encourage outside activity during shelter in place measures.” A father, with a household income of < $45K, stated that his son is “riding his hoverboard, playing with the dogs, or jumping on the trampoline.” We did not observe any differences between the two income groups of our sample in terms of how much equipment or what type of equipment children had access to. When parents shared that their children had access to equipment, they also usually described their children as active.

### Parental Support of Physical Activity

The fourth theme was that *parents support their children's physical activity*. Parents showed support for their children's PA through their involvement and encouragement. Parents were involved in their children's PA by playing with them outside, exercising indoors together, or taking the family on walks, hikes, or bike rides. Parents encouraged their children to be active by reminding them to play outside, providing equipment, and giving access to online resources such as Pokémon Go or YouTube workouts. A father of two boys with a household income >$120K stated, “We have as a family picked up bike riding, taking the dogs for daily walks, basketball in the driveway along with four square, swimming, and tennis.” A mother of a 12-year-old girl, with a household income of < $45K, shared, “We use my exercise bike for 10 min each day, 10 more minutes of yoga, and multiple dance parties each day to help break up the monotony and stop anxiety when it starts.” This theme was found across all income brackets.

Some parents also mentioned being busy during the shelter-in-place mandates, and that their busyness was a barrier to their involvement in their children's PA. In the higher income group, a few parents reported being busier due to work obligations. A father of three boys, with a household income in the $70–95K range, said, “my wife and I are working from home and don't have much time to provide supervision.” There was only one mention of a parent being busy due to work obligations in the lower income bracket.

## Discussion

This study sought to understand, from the perspective of parents, how the COVID-19 shelter-in-place mandates affected children's physical activity, while also considering families' socioeconomic status. Results from this study showed that overall shelter-in-place mandates restricted children's opportunities for PA. Factors that facilitated children's PA included outdoor spaces and equipment. However, families in the lower income bracket had less access to outdoor spaces, when compared to families in the higher income bracket. Lastly, findings showed that despite the drastic changes brought on by the shelter-in-place mandates, parents continued to support and encourage their children's PA.

Data from parents make evident that children's PA opportunities were substantially limited because of shelter-in-place mandates. Specifically, parents reported that children could no longer participate in face-to-face PE classes, recess, sports, or after-school programs. Because families were socially distancing, children were unable to play with friends from schools or sport teams. The information gleaned by parents highlights the importance of outdoor spaces and equipment in facilitating children's PA. Upon examining this data by income bracket it became apparent that families in the lower income bracket had less access to outdoor play spaces when compared to families in the higher income bracket. Parents in the lower income bracket described restricted indoor home spaces that impeded children's PA. On the other hand, parents in the higher income bracket described private outdoor spaces, such as backyards. Finally, parents shared a plethora of ways that they supported children' PA. This included coordinating family activities (e.g., bike rides, hikes, and walks). Parents also encouraged and supervised PA, be it indoors or outdoors. Parents worked to increase children's PA by integrating virtual means, such as YouTube videos, Pokémon Go phone applications, or online physical education videos. When parents owned indoor workout equipment, such as treadmills or stationary bikes, they encouraged children to use that equipment as well.

The findings from this study align with previous research. In this current study, parents shared that children lacked peers to play with. Importantly, classmate social support has been shown to predict PA in children (18). Because of social distancing, children may have engaged in less PA, while simultaneously missing the valuable social context that sports, PE, recess, and other extracurricular programs provide. This study shed light on the discrepancy in access to outdoor spaces by income bracket. Similarly, other research shows that children in low-income communities have fewer opportunities to engage in PA, due in part to a lack of safe, outdoor green spaces (7, 8). Access to active outdoor play is a valuable component for healthy child development (19). The more time (20), outdoor space (21), and equipment (22) children have, the more physically active children tend to be. Finally, these findings align with past research showing that parental support is an important correlate of children's PA (23, 24).

### Strengths

This study provided timely insight into how children's PA changed during the shelter-in-place mandates. The open-ended question enabled an exploratory analysis of parents' responses grounded in a social constructionist approach to knowledge. Moreover, consideration of families' household income resulted in a nuanced understanding of how socioeconomic factors could influence children's PA.

### Limitations

The major limitation is that the primary data source came from a single open-ended question. Moreover, the answers provided by parents varied in depth. Some parents provided short replies, whereas others included more detail about how their children's PA has been affected. Shelter-in-place mandates varied across the USA, as every state released various guidelines at different times and stages. Furthermore, the sample from this study is not representative of the USA. Specifically, this research sample heavily skewed White, which may limit generalizability. Future research should be sure to include the perspectives of people of color and be representative of the population. Moreover, this sample was recruited *via* an online data collection platform (Prolific), which most likely differs from the general USA population, in terms of both demographics and psychographics. Finally, this study surveyed parents, but children may have a different perspective of how the shelter-in-place mandates affected their PA. Future studies could examine children's accounts of the shelter-in-place mandates.

### Recommendations

These findings can help inform health promotion interventions during the pandemic and beyond. The lack of outdoor space appeared to be most prohibitive of PA, and parents in the lower-income bracket had a greater need for access to safe outdoor spaces. To address this problem, schoolyard spaces (e.g., playgrounds, basketball courts, and fields) should be made available to families even when school is not in session. Importantly, these spaces should be safe, accessible, and well-maintained to facilitate children's PA. Moreover, not just schools, but local community centers, such as the Boys and Girls Club or YMCA, could continue to support families by making spaces available for children to use. Community stakeholders and policymakers should advocate for safe, equitable access to outdoor spaces by investing funds into maintaining and renovating schoolyards, playgrounds, and public parks. In conjunction with outdoor spaces, sport and play equipment were found to facilitate PA, and the lack thereof may potentially prohibit PA for low-income families. Schools and community organizations could loan play equipment to families for use at home or while on the schoolyard. The model for such a program could mirror current laptop loan programs.

Parents adopt greater responsibility and may require more support as they navigate PA at home during school closures, including scheduled summer and winter breaks (25). Parents of all income brackets can be given support about popular virtual PA-related tools, such as Pokémon Go or Go Noodle. Schools and community centers could share with parents how to effectively facilitate PA indoors (e.g., dance and yoga) and outdoors (e.g., handball and four-square). During shelter-in-place mandates, PE teachers and community coaches could offer virtual PE classes and PA-related lessons. After-school or sport programs could continue to run on schoolyards or at local community centers during summer and winter breaks to help relieve parents' responsibilities.

## Conclusion

The COVID-19 shelter-in-place mandates led to the closures of schools and public spaces, which limited children's PA opportunities. These findings demonstrate that parents strove to support their children's PA during the pandemic. Moreover, this study makes evident that equitable access to outdoor spaces and play equipment generally support children's PA. These findings can help inform public health policy geared toward supporting families of lower socioeconomic status with less access to safe outdoor spaces. Moreover, by identifying the factors that promote children's PA during the shelter-in-place mandates, this study also provided pertinent insight into factors that could enhance children's PA outside of the school environment more generally.

## Data Availability Statement

The raw data supporting the conclusions of this article will be made available by the authors, without undue reservation.

## Ethics Statement

The studies involving human participants were reviewed and approved by Oregon State University Human Research Protection Program and Institutional Review Board. The patients/participants provided their written informed consent to participate in this study.

## Author Contributions

DP: methodology, formal analysis, and writing—original draft. JT: investigation, formal analysis, and writing—original draft. NU**:** formal analysis and writing—original draft. CK: data interpretation and writing—reviewing and editing. WM: writing—reviewing and editing, data interpretation, and supervision. All authors contributed to the article and approved the submitted version.

## Conflict of Interest

The authors declare that the research was conducted in the absence of any commercial or financial relationships that could be construed as a potential conflict of interest.

## Footnotes

^1^For ease, the term parent is used throughout this paper to account for all participants.

## Appendix

### Parents of School-Aged Children Survey

**Questions**

1. Do you have children in elementary or secondary school (K–12)?
2. Number of children in elementary or secondary school (K–12)?
3. Please fill out age, grade, and gender for each child:
4. Relationship to child(ren)?
5. Parent (your) race/ethnicity?
6. What is your household income?
7. What is your primary occupation?
8. Do any of your children have any developmental disabilities (e.g., autism, down-syndrome, etc.)? If yes, please list:
9. Do any of your children have any physical disabilities? If yes, please list:
10. Do any of your children have any documented behavioral or emotional disabilities (e.g., ADHD, anxiety, depression, etc.)? If yes, please list:
11. Do any of your children qualify for free or reduced-price lunch at school?
12. Describe your children's level of physical activity during COVID-19 shelter-in-place mandates.
